# Transdiagnostic Cognitive-Behavioral Therapy for Depression and Anxiety Disorders in Cardiovascular Disease Patients: Results From the CHAMPS Pilot-Feasibility Trial

**DOI:** 10.3389/fpsyt.2022.741039

**Published:** 2022-04-14

**Authors:** Phillip J. Tully, Deborah A. Turnbull, John D. Horowitz, John F. Beltrame, Bernhard T. Baune, Shannon Sauer-Zavala, Harald Baumeister, Christopher G. Bean, Ronette B. Pinto, Suzie Cosh, Gary A. Wittert

**Affiliations:** ^1^Freemasons Centre for Male Health and Wellbeing, South Australian Health and Medical Research Institute, School of Medicine, The University of Adelaide, Adelaide, SA, Australia; ^2^School of Psychology, The University of Adelaide, Adelaide, SA, Australia; ^3^Department of Cardiology, Basil Hetzel Institute, The Queen Elizabeth Hospital, Adelaide, SA, Australia; ^4^Department of Psychiatry, University of Münster, Münster, Germany; ^5^Department of Psychiatry, Melbourne Medical School, The University of Melbourne, Melbourne, VIC, Australia; ^6^The Florey Institute of Neuroscience and Mental Health, The University of Melbourne, Parkville, VIC, Australia; ^7^Department of Psychology, University of Kentucky, Lexington, KY, United States; ^8^Department of Clinical Psychology and Psychotherapy, Institute of Psychology and Education, Ulm University, Ulm, Germany; ^9^School of Psychology, The University of New England, Armidale, NSW, Australia

**Keywords:** depression, major depression (MDD), anxiety, anxiety disorders, cardiovascular disease, coronary heart disease, randomized controlled trial, cognitive-behavioral therapy (CBT)

## Abstract

**Objective:**

The aim of the Cardiovascular Health in Anxiety and Mood Problems Study (CHAMPS) is to pilot the Unified Protocol (UP) for the transdiagnostic treatment of depression and anxiety disorders in patients recently hospitalized for cardiovascular diseases (CVDs) and evaluate the feasibility.

**Methods:**

The present study is a controlled, block randomized pragmatic pilot-feasibility trial incorporating qualitative interview data, comparing UP (n = 9) with enhanced usual care (EUC, *n* = 10). Eligible trial participants had a recent CVD-cause admission and were above the severity threshold for depression or anxiety denoted by Patient Health Questionnaire (PHQ-9) total scores ≥10 and/or Generalized Anxiety Disorder (GAD-7) total scores ≥7 respectively on two occasions, and met criteria for one or more depression or anxiety disorders determined by structured clinical interview. Study outcomes were analyzed as intention-to-treat using linear mixed models and qualitative interview data were analyzed with content analysis.

**Results:**

Quantitative and qualitative measured indicated acceptability of the transdiagnostic CBT intervention for CVD patients with depression or anxiety disorders. Satisfaction with UP was comparable to antidepressant therapy and higher than general physician counseling. However, there were difficulties recruiting participants with current disorders and distress on two occasions. The UP was associated with a reduction in total number of disorders determined by blinded raters. Linear mixed models indicated that a significantly greater reduction in anxiety symptoms was evident in the UP group by comparison to the EUC group (GAD-7, *p* between groups = 0.011; Overall Anxiety Severity and Impairment Scale, *p* between groups = 0.013). Results favored the UP group by comparison to EUC for change over 6 months on measures of physical quality of life and harmful alcohol use. There was no difference between the two groups on changes in depression symptoms (PHQ-9), stress, metacognitive worry beliefs, physical activity, or adherence.

**Discussion:**

In conclusion, this feasibility trial indicates acceptability of transdiagnostic CBT intervention for CVD patients with depression or anxiety disorders that is tempered by difficulties with recruitment. Larger trials are required to clarify the efficacy of transdiagnostic depression and anxiety disorder CBT in populations with CVDs and depressive or anxiety disorders.

**Clinical Trial Registration:**

https://www.australianclinicaltrials.gov.au/anzctr/trial/ACTRN12615000555550, identifier: ACTRN12615000555550.

## Background

Depressive disorders are prevalent in 10–15% of the population with cardiovascular diseases (CVD) such as coronary heart disease and heart failure (1, 2). Comorbid depression in patients with CVD portends an increased risk for morbidity and mortality (3, 4) and higher healthcare costs (5). To date, antidepressant and cognitive-behavioral therapy (CBT) interventions have resulted in significant reductions in depression symptoms, albeit inconsistent reductions on major adverse cardiac outcomes (MACE) (6, 7). One limitation of past interventions is the predominant focus on depression in isolation of other psychosocial risk factors. Psychosocial risk factors for CVD tend to cluster together within individuals or groups (8–10). These interrelated psychosocial factors include anxiety disorders, hostility, anger, stress, worry, rumination, anxiety sensitivity, phobic anxiety, distress tolerance, and the specific combination of negative affect and social inhibition (11–17). Anxiety disorders in particular are associated with onset of CVD and adverse cardiovascular prognosis (18–22). Moreover, anxiety disorders have high concurrent and lifetime comorbidity rates with depression disorders in the general population (23) and those with CVD (15, 24). Collectively, these findings point to the likelihood that common processes underlying negative emotions generally elevate CVD risk and *vice versa* (12). This raises the possibility that an intervention transcending diagnostic boundaries, targeting core emotional processes, would be a step toward improving mental health interventions amongst CVD populations.

Indeed, parallel trends in clinical psychology are exploring the tenet that CBT interventions transcending diagnostic boundaries may offer advantages over disorder-specific treatments (25, 26). For example, transdiagnostic interventions may be more appropriate in the common clinical situation of comorbid disorders, without the need to prioritize one disorder over another (i.e., selecting one single-disorder protocol over another) (27). To date, few studies have examined transdiagnostic CBT interventions for depression and anxiety disorders in CVD populations. A RCT of meta-cognitive therapy in cardiac rehabilitation patients showed that results favored the meta-cognitive therapy arm for reduction in Hospital Anxiety and Depression Scale total score at 4 and 12 months post-intervention compared to usual care (standardized effect size 0.52 and 0.33 respectively) (28). Moreover, transdiagnostic approaches have been successfully applied to other health conditions including functional gastrointestinal diseases (29, 30), cancer (31), HIV (32), headache (33).

The aim of the Cardiovascular Health in Anxiety and Mood Problems Study (CHAMPS) is to prospectively study the feasibility and acceptability of the Unified Protocol (UP) for the Transdiagnostic Treatment of Emotional Disorders (34) in patients with a recent CVD hospitalization and comorbid depression or anxiety disorder. A feasibility study is considered preliminary work undertaken to estimate important parameters necessary for the design of a larger clinical trial.

## Methods

### Study Design

This prospective study is a feasibility randomized controlled trial, of parallel design, comparing the UP for emotional disorders vs. enhanced usual care (EUC). A trial protocol was registered (ACTRN12615000555550) and summarized elsewhere (35). This study was approved by Human Research Ethics Committee of the Queen Elizabeth Hospital (Approval #HREC/15/TQEH47). A power calculation was not performed for this feasibility trial with the self-rated outcome measures collected to provide the standard deviation of patient outcomes which is necessary to perform a power calculation in a larger and more definitive trial.

### Study Population

Participant eligibility criteria were: (1) age ≥ 18 years; (2) a primary hospital admission for CVD (specified by relevant International Classification of Disease codes (36) for myocardial infarction, coronary revascularization, symptomatic coronary disease, heart failure, heart valve disease, atrial or ventricular arrhythmia); (3) above the threshold for depression and/or anxiety denoted by Patient Health Questionnaire (PHQ-9) total scores ≥10 (37) and/or Generalized Anxiety Disorder (GAD-7) total scores ≥7 (38) respectively on two occasions (index admission and 2 weeks later); and a MINI International Neuropsychiatric Interview (MINI) 5.0.0 diagnosis of any major depression ± melancholic features, dysthymia, panic disorder, agoraphobia, social anxiety disorder, generalized anxiety disorder (GAD) or post-traumatic stress disorder (PTSD); and (4) fluency in English. Participants who were no longer above the threshold for depression and anxiety symptoms on the PHQ-9 and GAD-7 at the second assessment were invited to continue their involvement in an observational sub-study as a non-distressed control group.

Participant exclusion criteria were: (1) psychosis or bipolar disorder determined by medical history or randomization naïve assessors; (2) high suicide risk; (3) cognitive impairment or dementia impeding delivery of psychotherapy or provision of informed consent; (4) neurodegenerative condition such as Parkinson's Disease; (5) receiving psychologist or psychiatrist counseling elsewhere or psychotropic treatment at enrollment; (6) a diagnosis of drug and alcohol dependence or abuse determined by randomization naïve assessors; (7) a medical condition likely to be fatal within 1 year.

**Figure 1:**
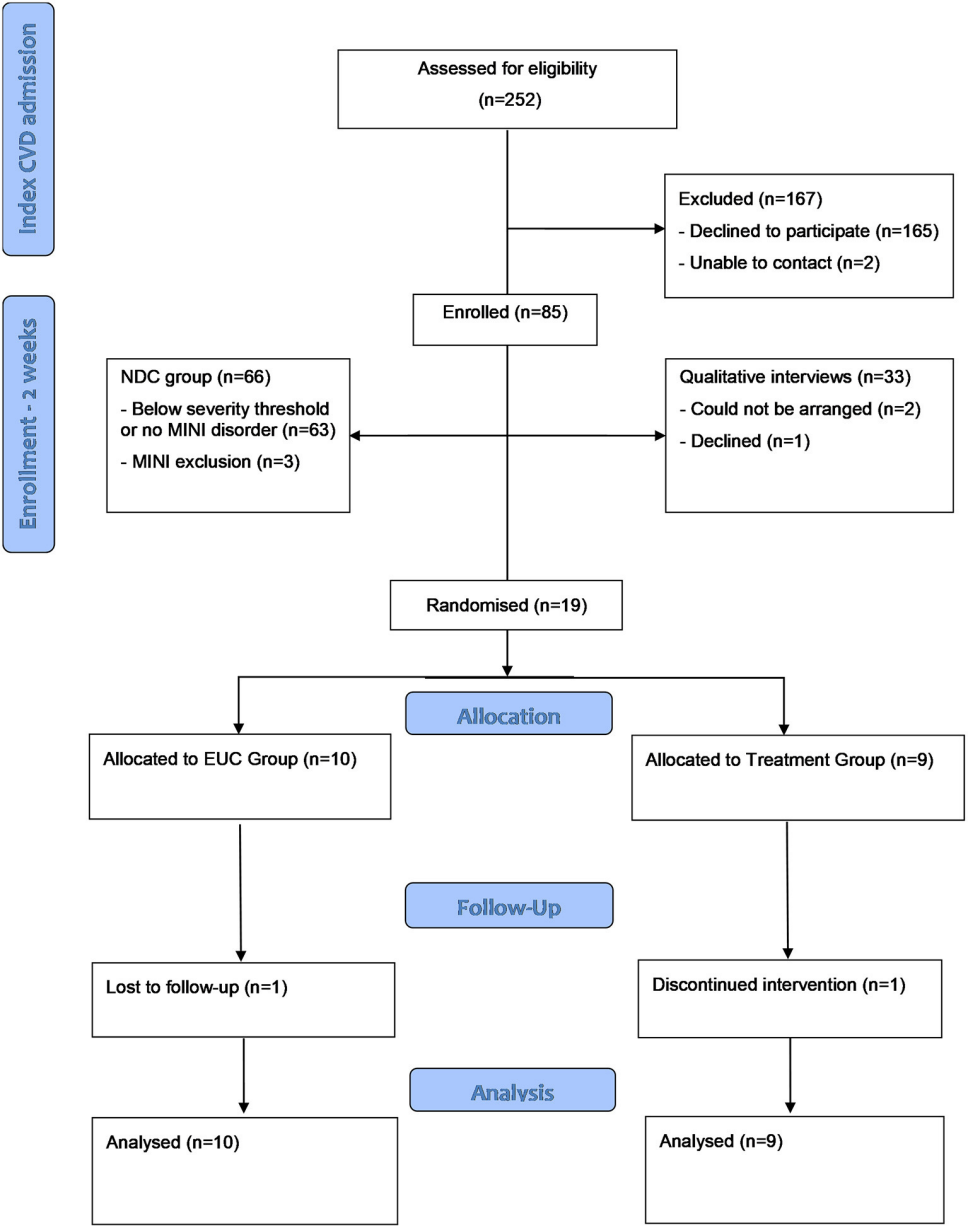
Flow chart of CHAMPS participants through the study. CVD, cardiovascular disease; CHAMPS, Cardiovascular health in Anxiety or Mood Problems Study; EUC, enhanced usual care; GAD-7, generalized anxiety disorder-7; MINI, MINI international Neuropsychiatric Interview; NDC, non-distressed control; PHQ, Patient Health Questionnaire; UP, unified protocol.

Participants were recruited from the Queen Elizabeth Hospital, a public hospital in the Western urban area of Adelaide, South Australia. Parallel to the main trial, a non-distressed control group was recruited, comprised of patients below the severity threshold for depression or anxiety denoted by PHQ-9 and GAD-7 2 weeks post-admission, or not meeting criteria for a depression or anxiety disorder specified by inclusion criterion #3 (Figure 1). The non-distressed control group were recruited to evaluate screening procedures (39) and determine longer-term symptom trajectories similar to previous trials (40), and are not described further here.

### Protocol Deviations

In contrast to the published protocol (35), participants were only included if they met criteria for a current depression and/or anxiety disorder and had elevated symptoms on two occasions, as opposed to the original criteria of elevated symptoms ± a depression or anxiety disorder. Consequently, randomization was not stratified by primary CVD-cause admission and the target recruitment of 50 patients was revised to 20 participants due to recruitment difficulties of severe mental disorders among the CVD population as other trials have noted (41). Also, during the roll-out phase, an amendment was made to include a questionnaire measuring meta-worry, along with a qualitative sub-study. During the ethics amendment process, trial recruitment was suspended as the ethics committee mandated, for all patients with a severe depressive symptoms in the control group (i.e., PHQ-9 scores ≥ 20) at any stage of the study, that a community based acute crisis mental health triage service was contacted as part of duty of care. Similarly, for any control patients with moderate to moderately severe depressive symptoms (i.e. PHQ scores ≥10 and ≤ 19) at any stage of the study, that the patient and their general physician be notified.

### Randomization and Masking

An independent statistician generated the randomization codes. Patients were block randomized according to a random number generator in alternating block sizes of four and six. After baseline measures were completed, an electronic and automated email was sent to the clinical trial manager containing a code for randomization to either the UP or EUC. In psychotherapy trials, the patients, therapist, and clinical trial manager are aware of treatment allocation. The research assistants who performed the assessments, data extraction and entry remained blind to treatment allocation through all stages of the study and follow-up.

### Transdiagnostic Unified Protocol Intervention

The UP is a type of CBT explicitly designed to address the full range of emotional disorders (anxiety, depressive, and related disorders) by targeting core and underlying emotional processes that lead to the development and maintenance of symptoms across disorders. The UP was designed by Barlow et al. for weekly and face-to-face delivery up to a maximum of 18 weeks (42). The UP comprises eight modules: (1) enhancing motivation for change and treatment engagement, (2) facilitating better understanding of patients' emotional experiences, (3) increasing present focused emotion awareness, (4) increasing cognitive flexibility, (5) identifying and preventing patterns of emotion avoidance and maladaptive emotion driven behaviors, (6) increasing awareness and tolerance of emotion-related physical sensations, (7) interoceptive and situation-based emotion focused exposure, and (8) the final module was devoted to summarizing the relevant techniques attained and developing relapse prevention strategies. Owing to the highly medicalized nature of treating anxiety and depression in CVD populations and patient preference for shorter therapies (43), during piloting we refined the UP from 18 sessions, covering 8 modules (and the preliminary module), down to 12 sessions, opting for 50 to 90 min weekly sessions (see eSupplement1 in Supplementary Material). An experienced psychologist (CGB), who was trained in both the manualized UP (42) and on common CVDs, delivered the intervention. Intervention fidelity was maintained by weekly supervision with a senior psychologist and discussion of cases.

### Enhanced Usual Care

Patients randomized to the EUC group received an education package which was delivered by the clinical trial manager, consisting of the beyondblue^®^ fact sheet regarding anxiety, depression and coronary heart disease (44), conforming with the National Heart Foundation of Australia's^®^ guidelines (37). Participants and their general physician were informed of the baseline depression severity results and directed to available clinical services (psychologist, psychiatrist, telephone counseling), advising participants to seek assistance for achieving mental wellbeing. As abovementioned, additional duty of care practices and general physician notification was implemented for patients with depressive symptoms at the request of the ethics committee. There were no other restrictions placed on usual care.

### Procedure

Potentially eligible patients were identified 1–4 days after the index CVD admission by the clinical trial manager in the cardiology department. Eligible and consenting participants underwent a basic screening procedure consisting of the PHQ-9 and GAD-7. Participants were re-assessed at 2 weeks post-index CVD admission to complete the PHQ-9, GAD-7, and a structured clinical interview to confirm symptom severity and study eligibility, as well as complete the baseline patient rated measures (see eSupplement 2 in Supplementary Material). After confirmation of study eligibility and completion of baseline study measures, the allocation was revealed to the clinical trial manager, and arrangements made for either the UP or EUC arm. Patients ineligible for the trial at 2 weeks due to low symptom severity on the PHQ-9 or GAD-7 were invited to participate in the non-distressed control group. The qualitative sub-study sampled equally from the UP, EUC, and non-distressed control group.

### Patient Rated Measures

The complete battery of measures and their timing of assessment is described in eSupplement 2 in Supplementary Material.

#### Generalized Anxiety Symptoms

The GAD-7 approximates Diagnostic and Statistical Manual 5 (DSM-5) criteria for GAD (45). The GAD-7 is scored on a scale of 0–3 (not at all, several days, more days than half the days, and nearly every day). The GAD-7 is considered valuable for use the detection of anxiety disorders in medical and primary care populations because the measure does not contain any somatic items (45). The GAD-7 severity threshold for clinically relevant symptoms are total GAD scores ≥ 7 (38) which has favorable validity to identify depression and anxiety disorders in medical patients (38).

#### Anxiety Severity

The Overall Anxiety Severity and Impairment Scale (OASIS) was developed as a self-report measure that assesses clinical severity and functional impairment of anxiety disorders (46, 47). Participants respond to 5 items that best describe their experience on a five-point scale (0, little or none; 1, mild; 2, moderate; 3, severe; 4, extreme). Scores >8 are indicative of severe anxiety in primary care and psychiatric samples (47).

#### Depression Symptoms

The PHQ-9 is a standardized instrument that incorporates DSM-5 major depression disorder criteria (48). Each item of the PHQ-9 is scored from 0 to 3, with scores ranging from 0 to 27. Scores of 10, 15, and 20 represent the thresholds for moderate-, moderately severe-, and severe-depression respectively (48). The PHQ-9 is recommended for depression screening in CVD populations because of its specificity to detect depression and sensitivity to clinical change (14, 37, 49).

#### Stress

The Depression, Anxiety and Stress Scales (DASS-21) is a 21 item measure validated in adults to age 90 years (50, 51). Scores range from 0 to 21 for the stress subscale with higher scores denoting higher stress symptoms. The DASS-21 factor structure approximates a tripartite structure, with stress broadly indicative of negative affectivity shared between depression and anxiety disorders (52).

#### Meta-Cognitions

The Metacognitions about Symptoms Control Scale (MaSCS) assesses metacognitive beliefs pertaining to symptoms in chronic health conditions (53). The MaSCS is a 17-item questionnaire, asking participants to rate on a 4-point Likert scale (1 = *Do not agree*, 4 = *Agree strongly*) how much a statement applies to them (e.g., “If I focus on the symptom, I can take the appropriate action to get better”). In line with conceptualizations of meta-worry, the MaSCS has two subscales, one each tapping into positive metacognitive worry beliefs and negative metacognitive worry beliefs. The MaSCS was developed for a variety of chronic conditions. The MaSCS instructions were adapted to state “*This scale is concerned with how people with cardiovascular disease experience and cope with their symptoms*.”

#### Quality of Life and Health Behaviors

Quality of life (QOL) was assessed with the 12-item short-form health survey (SF-12) (54). The SF-12 is one of the most commonly utilized and generalizable measures of QOL in general populations as well as CVD populations (55). The SF-12 sub-scale scores were arranged into the Physical Components Scale (PCS) and Mental Components Scale (MCS) in accordance with the manual. The PCS and MCS are weighted summary scales (range width 0–100) with higher scores representing higher QOL.

Behavioral factors such as alcohol use and smoking are pertinent to cardiovascular functioning, and are potential emotion-driven behaviors under the UP conceptualization of responses to anxiety and depression. Harmful alcohol use was measured by the Alcohol Use Disorders Identification Test Shortened Clinical (AUDIT-C) version which provides favorable sensitivity and specificity for the detection of problematic drinking (56). The physical activity questions from the Australian National Health Surveys was used to classify participants level of physical activity (57). Due to inconsistent reporting and missing data, it was not possible to calculate metabolic equivalents and thus only total activity (in minutes) is reported. Lifetime and current tobacco use was measured by items from the Global Adult Tobacco Survey (GATS) (58).

#### Adherence

Adherence was measured by 5 items from the Medical Outcomes Study Specific Adherence Scale (MOS SAS) (59). The items used in CHAMPS covered adherence to diet, exercise, stress management, cardiac rehabilitation and medication.

### Mental Disorders

The MINI version 5.0.0 is a brief structured interview to diagnose common depression and anxiety disorders as well as alcohol and substance abuse and psychosis as outlined in the inclusion and exclusion criteria (60, 61). The MINI modules utilized in this study covered; major depressive disorder, major depressive disorder with melancholic features, dysthymia, hypomanic and manic episode (bi-polar I and II), panic disorder, agoraphobia, social anxiety disorder, obsessive-compulsive disorder (OCD), PTSD, alcohol dependence/abuse, substance dependence/abuse (non-alcohol), psychotic disorders, mood disorders with psychotic features, GAD, and antisocial personality disorder. Specific phobias are not assessed by the MINI 5.0.0 and the modules relating to anorexia and bulimia nervosa were omitted. Assessments were performed to determine the mental health diagnosis at baseline and remission at the end of the study by qualified junior psychologist assessors who were blinded to treatment allocation and the timing of the assessment (e.g. pre-, post).

### Medical Outcomes, Intervention Acceptability and Feasibility, Qualitative Analyses

These methods are described in the eSupplement 3 in Supplementary Material.

### Statistical Analysis

For participants missing any self-reported data at 18 weeks or 6 months, data were imputed to increase statistical power using multiple imputation procedures based on demographic and medical comorbidities. All analyses were performed based on intention-to-treat. Comparisons between UP and EUC were made at baseline on demographics and clinical risk factors using independent samples *t*-tests, Kruskal–Wallis test, and the chi-square statistic with Fisher's exact test where appropriate. Continuous data were analyzed with linear mixed models to assess between UP and EUC group differences in the change of a given variable from baseline to 6 months follow-up (62). Mixed model data assumptions were met and each mixed model specified correlated residuals within subjects, random effects with restricted maximum likelihood function, and robust covariance estimation, utilizing a random slope and random intercept. Mixed model data are reported with M ± SD at each observation point to assist with effect size estimates in future studies or meta-analyses. For depression and anxiety disorder diagnoses at 6-month follow-up, the total number of disorders were tallied to calculate the change in number of baseline and follow-up disorders. For hospital readmissions, MACE and psychiatric admissions were analyzed with the chi-square statistic with Fisher's exact test. For satisfaction with care, only descriptive statistics are reported. As a feasibility trial and largely exploratory study to help inform the power calculations of a larger trial, no adjustment was made for multiple comparisons.

## Results

A total of 19 participants were randomized to either UP (*n* = 9) or EUC (*n* = 10). Two participants dropped out (1 from each arm) by 6 months. There were no differences between UP and EUC patients in any baseline demographic, cardiovascular or mental health variables (Tables 1, 2). The most common mental disorders at baseline were major depressive disorder, agoraphobia, and GAD. At baseline, 5 patients met criteria for 1 disorder (26.3%), 4 met criteria for 2 disorders (21.1%), 8 met criteria for 3 disorders (42.1%), and 2 met criteria for 4 disorders (10.5%).

**Table 1:** Baseline demographic and cardiovascular characteristics of the sample *N* (%).

	**Enhanced usual care (*N* = 10)**	**CBT-Unified Protocol (*N* = 9)**	***P***
Median age, years (IQR)^a^	62 (55–72)	62 (41–72)	0.39
Male sex	5 (50.0)	4 (44.4)	0.99
**Employment status**			0.87
Currently employed	5 (50.0)	4 (44.4)	
Retired	4 (40.0)	5 (55.6)	
Unemployed	1 (10.0)	-	
Current tobacco smoking	1 (10.0)	1 (11.1)	0.99
**Index-CVD admission**			
Acute myocardial infarction	1 (10.0)	2 (22.2)	
Heart failure	2 (20.0)	3 (33.3)	
Arrhythmia	3 (30.0)	3 (33.3)	
Angina pectoris	4 (40.)	1 (22.2)	
**CVD comorbidities**			
Previous revascularization	2 (20.0)	-	0.47
Previous myocardial infarction	2 (20.0)	1 (11.1)	0.99
Valvular disease	1 (10.0)	1 (11.1)	0.99
Biventricular pacemaker	1 (10.0)	1 (11.1)	0.99
Implanted cardiac defibrillator	1 (10.0)	1 (11.1)	0.99
Prior stroke or TIA	2 (20.0)	–	0.47
Hypertension	8 (80.0)	5 (55.6)	0.35
Dyslipidemia	5 (50.0)	5 (56.6)	0.99
**Other pertinent comorbidities**			
Lung disease	2 (20.0)	1 (11.1)	0.99
Renal disease	2 (20.0)	–	0.47
Diabetes	2 (20.0)	1 (11.1)	0.99
BMI, kg/m^2^ ≥ 30	5 (50.0)	5 (55.6)	0.99
Sleep apnea	1 (10.0)	2 (22.2)	0.58
Chronic pain	4 (40.0)	1 (11.1)	0.30

a *All data presented as N(%) unless otherwise indicated.*

*BMI, body mass index; CBT, cognitive-behavioral therapy; CVD, cardiovascular disease; IQR, interquartile range; TIA, transient ischemic attack*.

**Table 2:** Baseline mental health characteristics of the sample.

	**Enhanced usual care (*N* = 10)**	**CBT-unified protocol (*N* = 9)**	***P***
**MINI diagnoses**			
Major depressive disorder	7 (70.0)	9 (100.0)	0.21
Depressive disorder with melancholic features^a^	5 (50.0)	4 (44.4)	0.99
Panic disorder	4 (40.0)	1 (11.1)	0.30
Agoraphobia	4 (40.0)	2 (22.2)	0.63
Social Anxiety Disorder	1 (10.0)	-	0.99
Generalized anxiety disorder	2 (20.0)	4 (44.4)	0.35
Obsessive compulsive disorder	–	1 (11.1)	0.47
Post-traumatic stress disorder	–	1 (11.1)	0.47
**Number of disorders on MINI** ^b^			0.76
1	3 (30.0)	2 (22.2)	
2	2 (20.0)	2 (22.2)	
3	4 (40.0)	4 (44.4)	
4	1 (10.0)	1 (11.1)	
**Psychological symptoms at index admission**			
PHQ-9 score	12.6 ± 6.2	13.4 ± 4.6	0.74
GAD-7 score	9.5 ± 3.3	10.7 ± 5.6	0.59

a *Patients must also meet criteria for major depressive disorder.*

b *Inclusive of obsessive compulsive disorder which was not an eligibility disorder.*

*CBT, cognitive-behavioral therapy; GAD-7, Generalized Anxiety Disorder 7-item scale; MINI, Mini International Neuropsychiatric Interview; PHQ-9, Patient Health Questionnaire 9-item scale*.

### Psychological and QOL Outcomes

The number of disorders determined by blinded MINI raters was significantly lower in the UP group by comparison to the EUC (Table 3). Linear mixed models indicated that a significantly greater reduction in anxiety symptoms was evident in the UP group by comparison to the EUC group on the GAD-7 (*p* between groups = 0.011) and also the Overall Anxiety Severity and Impairment Scale (*p* between groups = 0.013). There was no difference between the UP and EUC groups on change in depression symptoms (PHQ-9), stress (DASS-21) or metacognitive worry beliefs. There was evidence favoring the UP group for greater improvement over 6 months in physical QOL, but not mental QOL.

**Table 3:** Change in psychological outcomes for patients randomized to unified protocol vs. enhanced usual care.

		**Control *N* = 10**		**UP *N* = 9**		***P* between groups**
		***M* (SD)**	**Δ (SD)**	**Pre *M* (SD)**	**Δ (SD)**	
MINI disorders	Pre	2.30 (1.06)	–	2.44 (1.02)	–	0.009*
	Post	1.00 (1.10)	−1.30 (0.48)	0.5 (1.00)	−1.94 (0.45)	
	6 month	3.00 (2.16)	0.70 (1.46)	1.40 (1.14)	−1.04 (0.69)	
Anxiety (GAD-7)	Pre	9.50 (3.31)	–	10.67 (5.57)	–	0.011*
	Post	3.30 (5.16)	−6.20 (3.21)	1.67 (2.78)	−9.00 (3.74)	
	6 month	4.65 (6.61)	−4.85 (4.43)	4.51 (3.66)	−6.16 (3.43)	
Anxiety impairment and avoidance (OASIS)	Pre	4.32 (3.95)	–	4.11 (4.82)	–	0.013*
	Post	1.80 (3.91)	−2.52 (2.48)	0.22 (0.67)	−3.89 (4.30)	
	6 month	2.89 (3.52)	−1.43 (2.39)	1.30 (2.21)	−2.81 (3.32)	
Depressive symptoms (PHQ-9)	Pre	12.60 (6.22)		13.44 (4.59)		0.16
	Post	5.81 (7.99)	−6.79 (4.80)	4.03 (5.99)	−9.41 (3.60)	
	6 month	8.97 (10.16)	−3.63 (6.39)	7.67 (6.40)	−5.77 (3.88)	
Stress (DASS-stress)	Pre	4.76 (5.44)	–	6.11 (7.64)	–	0.59
	Post	2.60 (6.65)	−2.16 (3.99)	0.78 (1.39)	−5.33 (6.58)	
	6 month	2.60 (6.65)	−3.86 (4.08)	0.78 (1.39)	−2.44 (4.58)	
Positive metacognitive worry beliefs (MaSCS)	Pre	16.00 (14.68)	–	21.33 (8.76)	–	0.95
	Post	4.40 (10.01)	−11.60 (8.98)	8.56 (14.52)	−12.78 (9.17)	
	6 month	9.10 (14.88)	−6.90 (9.35)	15.44 (14.92)	−5.89 (9.50)	
Negative metacognitive worry beliefs (MaSCS)	Pre	13.60 (12.39)	–	19.00 (9.19)	–	0.17
	Post	3.00 (6.75)	−10.60 (8.08)	3.00 (4.58)	−16.00 (5.45)	
	6 month	5.70 (10.11)	−7.90 (7.44)	9.11 (9.87)	−9.89 (6.06)	
Quality of life—(SF-12 PCS)	Pre	40.00 (5.10)	–	38.99 (8.67)	–	0.023*
	Post	42.23 (5.88)	2.23 (2.57)	47.74 (8.48)	8.75 (3.84)	
	6 month	43.18 (10.97)	3.18 (7.54)	46.17 (11.83)	7.18 (7.14)	
Quality of life—(SF-12 MCS)	Pre	35.47 (7.06)	–	34.80 (8.34)	–	0.59
	Post	37.84 (11.13)	2.37 (5.68)	41.60 (6.70)	6.80 (3.72)	
	6 month	40.49 (8.17)	5.02 (4.93)	40.41 (9.84)	5.61 (5.92)	

*CI, confidence interval; DASS, Depression Anxiety and Stress Scales; GAD-7, Generalized Anxiety Disorder 7-item scale; MaSCS, Metacognitions about Symptoms Control Scale; OASIS, Overall Anxiety Severity and Impairment Scale; PHQ-9, Patient Health Questionnaire 9-item scale; UP, Unified Protocol for the Transdiagnostic Treatment of Emotional Disorders. p < 0.05*.

### CVD Health Behaviors and Adherence

The CVD health behavior and adherence data are reported in Table 4. Linear mixed models indicated that a significantly greater reduction in harmful alcohol was evident in the UP group by comparison to the EUC group on the AUDIT-C. There were no differences between the UP group by comparison to the EUC group in physical activity or adherence. There were only two current smokers in the study and therefore GATS data was not analyzed.

**Table 4:** Change in health behaviors and adherence for patients randomized to UP.

		**Control (*N* = 10)**		**UP (*N* = 9)**		***P***
		**M (SD)**	**Δ (SD)**	**Pre M (SD)**	**Δ (SD)**	
Physical activity, minutes	Pre	16.00 (13.42)	–	15.71 (9.25)	–	0.15
	Post	35.00 (17.00)	19.00 (7.64)	43.33 (12.38)	27.62 (5.72)	
	6 month	43.00 (19.33)	27.00 (11.78)	53.00 (15.56)	37.29 (9.87)	
Alcohol use (AUDIT-C)	Pre	2.10 (3.25)	–	3.44 (3.58)	–	0.011*
	Post	3.10 (2.64)	1.00 (1.44)	2.11 (2.93)	−1.33 (1.58)	
	6 month	2.60 (1.65)	0.50 (2.17)	1.67 (2.29)	−1.77 (2.22)	
Adherence (MOS-SAS)	Pre	2.61 (1.67)	–	2.78 (1.44)	–	0.35
	Post	2.80 (1.13)	0.19 (0.82)	3.30 (1.21)	0.52 (0.63)	
	6 Month	3.67 (1.46)	1.06 (1.01)	3.44 (1.67)	0.66 (1.00)	

*AUDIT-C, Alcohol Use Disorders Identification Test-Shortened Clinical version; MOS-SAS, Medical Outcomes Study Specific Adherence Scale. p < 0.05*.

### Hospital Admissions

Hospital admissions for CVD causes at 6 months follow-up were comparable (UP 20.0% vs. EUC 22.2%, *p* = 0.99). One psychiatric admission occurred in the study (UP group 11.1% vs. EUC 0%, *p* = 0.47). This admission occurred in the context of concerns over cognitive function and personality change that was subsequently identified as a transient ischemic attack.

### Satisfaction With Care

Participants randomized to UP reported high satisfaction with the psychologist delivered UP intervention, median satisfaction = 4 “quite a bit satisfied,” and low satisfaction with psychological care provided by their GP, median = 1 “not at all satisfied.” However, EUC participants reported equally high satisfaction with antidepressants, median satisfaction = 4 “quite a bit satisfied” and equally low satisfaction with GP psychological care, median = 1 “not at all satisfied.”

### Qualitative Analysis

The CVD participants discussed numerous factors that contributed to their satisfaction or dissatisfaction with care. These were categorized into two overarching themes: characteristics of the treatment and of health professionals. Pertaining to CVD treatment, themes included identifying the problem and fixing it. Hence, accurate diagnoses and a successful outcome led to improvements in CVD symptom severity. By contrast, psychological treatment under the UP related to reducing severity, which impacted on overall satisfaction with medical care. With regard to medical professionals in general, including the psychologist, two main themes were developed. Firstly, the practitioner's personal characteristics which included subthemes of competence, efficiency, and personality. Competence was discussed in association with knowledge and expertise. Finally, being caring, helpful, and kind were crucial elements of practitioners' personal characteristics. The second theme pertaining to medical professionals was the practitioner-patient interaction. An important subtheme was quality of communication as perceived by patients, specifically—as mentioned by nearly all participants—receiving comprehensive information. Taken altogether, for patients to be satisfied, professionals must not only be competent (i.e., have knowledge and expertise), but also communicate effectively and explain information in a caring, helpful, and kind manner. Respect for the patient was identified as a subtheme that overlapped across practitioners' personal characteristics and practitioner-patient interactions.

## Discussion

This feasibility RCT showed that the UP intervention for emotional disorders was associated with greater change, by comparison to the EUC, on the total number of psychiatric disorders, anxiety, harmful alcohol use, and improvement in physical QOL. The UP was not associated with reductions in depression, stress, or metacognitive worry about symptoms. Likewise, the UP was not associated with changes in pertinent health behaviors such as physical activity level, adherence, or major hospitalization rates. As a feasibility study, the trial was not powered to detect significant clinical effects, thus all results need to be interpreted as preliminary. Moreover, there were difficulties with recruiting CVD patients who met criteria for current disorders and were distressed after the index admission which points to low feasibility of an outpatient intervention for patients with severe mental health concerns.

This study is perhaps the first CBT intervention in CVD populations to target anxiety disorders as part of its inclusion criteria. The current study indicated that the UP was associated with reductions in GAD-7 and OASIS scores, as well as cumulative number of disorders as adjudicated by blinded raters. The findings are highly significant because less is known about the treatment of anxiety disorders in CVD populations. Given that access to community mental health services is often limited to persons with a verified mental disorder (e.g., better access initiatives in Australia and Improving Access to Psychological Therapies in UK) (63, 64), the study findings support the referral of these patients with comorbid CVDs to mental healthcare professionals for transdiagnostic CBT. Indeed, as little empirical data currently exists pertaining to anxiety disorder treatment in CVD populations, it remains largely unknown whether transdiagnostic approaches are equivalent to single-disorder protocols in general (65) and in chronic diseases specifically (66). Recently, the UP has been tested in diverse health conditions including functional gastrointestinal diseases (30), cancer (31), HIV (32), and headache (33). The UP was particularly effective for emotional disorder and disease-related symptoms across multiple measures in functional gastrointestinal diseases (30), cancer (31), and HIV populations (32). Not all findings concerning the UP have been supportive, with this type of CBT found to be less effective across multiple outcomes among youth with anxiety disorders and headache (33). Similarly, in the present study there was no evidence of significant difference by comparison to EUC on depression, adherence, stress, metacognitive worry, physical activity, or adherence. The findings may raise preliminary questions of the suitability of UP to certain health conditions, or alternatively point to the need for refinements in intervention delivery and the results of larger more definitive trials.

One clinical implication of these findings is that the treatment of depressive symptoms and mental QoL in CVD may require a treatment approach other than the UP or in combination with other interventions. A recent Cochrane review (10) showed that both psychotherapy and pharmacological interventions have moderate to large effects on end of treatment depression scores though RCT evidence on the impact upon QoL remains sparse. Emerging data suggests that the sequential delivery of pharmacotherapy in the acute phase of depression and a short-term course of cognitive-behavioral therapy in the residual phase may be a suitable approach (67). Furthermore, the combination of CBT with other therapies such as wellbeing therapy could be optimal to enhance psychological wellbeing and improve quality of life (68, 69).

There is limited use of transdiagnostic interventions in CVD populations however past interventions have utilized collaborative care to treat depression and anxiety symptoms simultaneously in CVDs. For example, the Management of Sadness and Anxiety in Cardiology (MOSAIC) RCT (70) screened patients with the PHQ-9, GAD-7, and a panic-attack questionnaire to enroll participants into a multicomponent collaborative care intervention. Contrasting to the current findings, the MOSAIC trial showed that participants exposed to the intervention displayed significant reductions in depressive but not anxiety symptoms (70). A possible explanation is that the EUC group here were provided with enhanced monitoring at the recommendation of the ethics committee, though large trials have called into question the effectiveness of depression screening in patients with CVD (71). An alternative explanation is that the UP intervention group consisted of patients with GAD, panic disorder, agoraphobia, social anxiety disorder, OCD and PTSD, whereas the MOSAIC trial did not report anxiety disorder prevalence other than for GAD-7 symptom severity and responses to the panic attack screening question.

CBT may lead to improvements in health behaviors and adherence pertinent to CVD. A past CBT intervention combined with motivational interviewing as part of cardiac rehabilitation led to improvements in physical activity, dietary fat intake, medication adherence, and smoking abstinence (72). Here there was only a reduction in harmful alcohol intake, thus paralleling a transdiagnostic CBT intervention for eating disorders that led to reductions in alcohol intake (73). Concomitant reductions in harmful alcohol intake are theorized to relate to the UP's modules covering emotion-driven behaviors, which may include alcohol and illicit drug intake, smoking, and binge-eating behaviors pertinent to cardiovascular health (27). However, we emphasize that there is insufficient data from our trial to support the UP as a means to target binge-eating and smoking.

Participants randomized to UP reported high satisfaction with the psychologist delivered CBT intervention though this level of satisfaction was comparable to the EUC groups rating of satisfaction with antidepressants. In both cases, satisfaction with UP or antidepressants was higher than general physician counseling for mental health. The qualitative interviews suggested that medical and psychological care was viewed as related to the practitioner's personal characteristics, which included subthemes of competence, efficiency, and personality. Respect for the patient was identified as a subtheme that overlapped across practitioners' personal characteristics and practitioner-patient interactions. However, these themes likely parallel the therapeutic alliance, which is not specific to any particular psychotherapy, whether UP or otherwise.

This study is presented with several strengths including use of a manualized and transdiagnostic CBT intervention, blinded outcome ratings, and use of qualitative interviews to determine acceptability. There are several limitations of this feasibility trial including changes to the study protocol and difficulties recruiting similar to other recent trials in the CVD population (41), which points toward low feasibility for outpatient, face-to-face, and individual therapy. Consequently, a small sample size was obtained, thereby precluding definitive conclusions on UP treatment efficacy in CVD populations. Also, the small sample size precludes stratification of the findings by sex or age. The findings will require replication in adequately powered samples and potentially more homogenous samples such as post-acute coronary syndromes where depression treatment effect sizes are well characterized. The UP intervention is designed for multiple emotional disorders, likely resulting in heterogeneity in study participants' treatment needs that may be less related to the index CVD admission. For example, though depression and anxiety frequently occur in CVD populations, it is perhaps less likely that disorders such as social anxiety disorder would result from a CVD admission. A related limitation is that only 1 patient met criteria for PTSD thus it is unknown if the UP could be extended to PTSD in CVD populations. This is an important limitation to reconcile in future research as there are few RCT interventions for PTSD in CVD populations (74).

Another limitation of this study is that implementation of usual care was not possible, and the EUC design likely resulted in a level of monitoring and treatment greatly exceeding usual depression care (75). The increased intervention in EUC could potentially explain the lack of difference in PHQ-9 scores between UP and EUC groups. This somewhat parallels the findings of the 2 × 2 factorial CREATE trial where interpersonal therapy was not superior over clinical management in terms of depression outcomes (76). Other limitations concern the procedural challenges during the roll-out and intervention phase which included recruitment postponement, as well as staff unavailability and turnover which may have contributed to acceptability, attrition, and contaminate the feasibility of the intervention. Finally, the follow-up was limited to 6 months after the intervention period and longer-term follow-up would be required to examine durability of the intervention and the impact on CVD events and psychiatric readmissions.

In conclusion, our feasibility trial indicates acceptability of transdiagnostic CBT intervention for CVD patients with depression or anxiety disorders. The feasibility trial of UP intervention suggested superiority over enhanced usual care regarding the total number of disorders, anxiety symptoms, alcohol intake, and improvements in physical QOL. CVD patients with depression and anxiety disorders however may not benefit from UP with regards to depression, mental QOL, adherence, physical activity or hospitalization rates. Satisfaction with UP was comparable to antidepressant therapy and higher than general physician counseling. Future trials of the UP in CVD populations are warranted to confirm if treatment benefits are restricted to anxiety symptoms or might extend to depressive symptoms.

## Data Availability Statement

The raw data supporting the conclusions of this article will be made available by the authors, without undue reservation.

## Ethics Statement

The studies involving human participants were reviewed and approved by Human Research Ethics Committee of the Queen Elizabeth Hospital (Approval #HREC/15/TQEH47). The patients/participants provided their written informed consent to participate in this study.

## Champs Investigators

Linley A. Denson^1^, Megan Grech^1^, Marilyn Black^2^, Peter Cheung^2^, Alisson Barret^3^, Nathan Harrison^3^, Terina Selkow^2^ and Elizabeth Markwick^4^

^1^School of Psychology, University of Adelaide, Adelaide, SA, Australia; ^2^Department of Cardiology, Queen Elizabeth Hospital, Woodville, SA, Australia; ^3^Freemasons Foundation Centre for Men's Health, The University of Adelaide, Adelaide, SA, Australia; ^4^Department of Psychiatry, Queen Elizabeth Hospital, Woodville, SA, Australia.

## Author Contributions

PT, DT, JH, JB, BB, SS-Z, HB, and GW: study design. CB and RP: data collection. PT and HB: drafting manuscript. PT, DT, JH, JB, BB, SS-Z, HB, CB, RP, SC, and GW: critical revision of manuscript. All authors contributed to the article and approved the submitted version.

## Funding

This work was supported by the National Heart Foundation of Australia Vanguard Grant (100593) and the Menzies Foundation Allied Health Scholars Grant (AHS_004). PT was supported by the National Health and Medical Research Council of Australia (Neil Hamilton Fairley—Clinical Overseas Fellowship #1053578).

## Conflict of Interest

Advisory Board—Lundbeck, Janssen-Cilag; Grant/Research Support—AstraZeneca; Sanofi, Lundbeck; Honoraria—AstraZeneca, Bristol-Myers Squibb, Lundbeck, Pfizer, Servier Laboratories, Wyeth Pharmaceuticals, Takeda, Janssen, LivaNova PLC. The remaining authors declare that the research was conducted in the absence of any commercial or financial relationships that could be construed as a potential conflict of interest.

## Publisher's Note

All claims expressed in this article are solely those of the authors and do not necessarily represent those of their affiliated organizations, or those of the publisher, the editors and the reviewers. Any product that may be evaluated in this article, or claim that may be made by its manufacturer, is not guaranteed or endorsed by the publisher.
