# Supplementary material for: Transdiagnostic Cognitive-Behavioral Therapy for Depression and Anxiety Disorders in Cardiovascular Disease Patients: Results From the CHAMPS Pilot-Feasibility Trial
Source: Front Psychiatry. 2022 Apr 14;13:741039. doi: 10.3389/fpsyt.2022.741039 (PMC9050199; doi:10.3389/fpsyt.2022.741039)
Supplement: Supplementary file 1 [file Data_Sheet_1.DOCX]

**eSupplement 1. Outline of the Unified Protocol modules tailored to cardiovascular patients**

| Week | Module | Focus | Content |
| --- | --- | --- | --- |
| Week | Module |  |  |
| 1 | Preliminary, 1-I | Introduction | Introduction to emotional disorders, cognitive-behavioral therapy treatment format and procedures including monitoring, goal setting |
| 2 | 1-II | Motivation enhancement for treatment engagement | Focusing on enhancing motivation and readiness for change and treatment engagement |
| 3 | 2-I & 2-II | Psychoeducation | Understanding emotions, recognizing and monitoring emotional responses |
| 4 | 3-I | Emotion awareness training | Learning to observe emotional experiences and mindfulness |
| 5 | 3-II | Emotion awareness training | Learning to observe emotional experiences and mindfulness |
| 6 | 4-I & 4-II | Cognitive appraisal and reappraisal | Increasing cognitive flexibility |
| 7 | 5-I & 5-II | Emotion avoidance | Identifying and preventing patterns of emotion avoidance and maladaptive emotion-driven behaviors |
| 8 | 6 | Awareness and tolerance of physical sensations | Increasing awareness and tolerance of emotion-related physical sensations |
| 9 | 7 | Interoceptive and situational emotion exposures | Interoceptive and situation-based emotion focused exposure |
| 10 | 7 | Interoceptive and situational emotion exposures | Interoceptive and situation-based emotion focused exposure |
| 11 | 7 | Interoceptive and situational emotion exposures | Interoceptive and situation-based emotion focused exposure |
| 12 | 8 | Accomplishments, maintenance and relapse prevention | Summarizing the relevant techniques attained and developing relapse prevention strategies. |

**eSupplement 2. Assessment schedule for participants through the study**

|  |  |  | **Timing of Assessment for all participants** | | | | | |
| --- | --- | --- | --- | --- | --- | --- | --- | --- |
|  |  | **Eligibility** | **Pre**  **Allocation** | **4 weeks** | **8 weeks** | **12 weeks** | **18 weeks** | **6 months** |
| Variable | **Measure** |  |  |  |  |  |  |  |
| Inclusion criteria |  |  |  |  |  |  |  |  |
| Depression | PHQ-9 | ✓ |  | ✓ | ✓ | ✓ | ✓ | ✓ |
| Generalized Anxiety | GAD-7 | ✓ |  | ✓ | ✓ | ✓ | ✓ | ✓ |
| Psychiatric diagnosis | MINI | ✓ |  |  |  |  | ✓ | ✓ |
| Post-Eligibility |  |  |  |  |  |  |  |  |
| Anxiety Severity | OASIS |  | ✓ |  |  |  | ✓ | ✓ |
| General Stress | DASS-21 |  | ✓ |  |  |  | ✓ | ✓ |
| Positive metacognitive worry beliefs | MaSCS |  | ✓ |  |  |  | ✓ | ✓ |
| Negative metacognitive worry beliefs | MaSCS |  | ✓ |  |  |  | ✓ | ✓ |
| Quality of life | SF-12 |  | ✓ |  |  |  | ✓ | ✓ |
| CVD rehospitalization outcome | MACE |  |  |  |  |  | ✓ | ✓ |
| Exercise | Physical Activity |  | ✓ |  |  |  | ✓ | ✓ |
| Tobacco use | GATS |  | ✓ |  |  |  | ✓ | ✓ |
| Alcohol use | AUDIT-C |  | ✓ |  |  |  | ✓ | ✓ |
| Adherence | MOS SAS |  | ✓ |  |  |  |  |  |
| Psychiatric service, medication usage, satisfaction with care | Self-report, audit |  | ✓ |  |  |  | ✓ | ✓ |

*AUDIT-C, Alcohol Use Disorders Identification Test-Shortened Clinical version; CVD, cardiovascular disease; DASS-21, Depression, Anxiety Stress Scales; GAD-7, Generalized Anxiety Disorder-7; GATS, Global Adult Tobacco Survey; MACE, major adverse cardiac event; MINI, MINI International Neuropsychiatric Interview; MOS SAS, Medical Outcomes Study Specific Adherence Scale; OASIS, Overall Anxiety Severity And Impairment Scale; PHQ-9, Patient Health Questionnaire-9; SF-12, Medical Outcomes Study Short Form-12; QOL, quality of life;*

**eSupplement 3. Supplemental methods**

*Medical outcomes*

A state-wide registry of public hospital emergency, in- and out-patient contacts was used to identify CVD and psychiatric hospital admissions at 6 months follow-up. Major CVD events include myocardial infarction, stroke, coronary revascularization, cardiac failure and arrhythmia determined with relevant International Classification of Disease Criteria Codes I00-I99 [1]. Admissions for primary psychiatric causes include suicide attempt, deliberate self-harm or emergency department visit for panic disorder (International Classification of Disease Criteria Codes F00-F99) [1]. Electronic data linkage was used to determine admissions and adjudication of study outcomes was performed by an independent panel, blinded to randomization arm.

*Intervention acceptability and feasibility*

Participants were asked to rate how satisfied they were with different mental health care treatments that they had received, specifically; psychologist, psychiatrist, GP counselling, anti-depressant, and anti-anxiety medications (rating from 1 “not at all satisfied” to 5 “extremely satisfied”). Embedded within the main trial was a qualitative study examining participant perceptions of the quality of care received. The qualitative questions were designed to cover both medical and psychological care, and were collected though brief voluntary semi-structured telephone interviews and followed the consolidated criteria for reporting qualitative research guidelines [2]. Qualitative interviews sampled from the UP (n=8) and EUC groups (n=9) and NDC group (n=16).

*Qualitative interviews and analysis*

Interviews were transcribed according to Braun and Clarke's notation system [3]. Qualitative data were analyzed according to the stages of content analysis outlined by Braun and Clarke [3]. At the end of each interview, participants had the option of reviewing their transcript for correction. Although 17 participants (57%) requested a copy of their transcript, none made any corrections. Data saturation was reached by the 28^th^ interview. Data was initially coded by adopting a complete data-derived coding process; similar codes were subsequently grouped together to produce a final code. All relevant extracts for each code were collated. Three transcripts (10% of the data) were randomly selected, and comprehensive instructions were provided, for independent coding by a second qualitative researcher with relevant experience. All coding disparities and emergent findings were discussed in depth until consensus. The data were then revisited and re-analyzed based on consensus. Patterns in the data were developed into overarching themes, themes, and subthemes for satisfaction with care.

**eSupplement 4. Online references**

[1] World Health Organization, International Statistical Classification of Diseases and Related Health Problems 10th Revision Version for 2007 Available online at <http://www.who.int/classifications/apps/icd/icd10online/>, 2007.

[2] A. Tong, P. Sainsbury, J. Craig, Consolidated criteria for reporting qualitative research (COREQ): a 32-item checklist for interviews and focus groups, International Journal for Quality in Health Care 19(6) (2007) 349-357.

[3] V. Braun, V. Clarke, Successful qualitative research: a practical guide for beginners, SAGE Publications Ltd, Croydon, UK, 2013.
